# Association between Air Pollution and Suicide in South Korea: A Nationwide Study

**DOI:** 10.1371/journal.pone.0117929

**Published:** 2015-02-18

**Authors:** Youngdon Kim, Woojae Myung, Hong-Hee Won, Sanghong Shim, Hong Jin Jeon, Junbae Choi, Bernard J. Carroll, Doh Kwan Kim

**Affiliations:** 1 Department of Psychiatry, Samsung Medical Center, Sungkyunkwan University School of Medicine, Seoul, Korea; 2 Samsung Biomedical Research Institute, Seoul, Korea; 3 Pacific Behavioral Research Foundation, Carmel, California, United States of America; NSYSU, TAIWAN

## Abstract

Suggestive associations of suicide with air pollutant concentrations have been reported. Recognizing regional and temporal variability of pollutant concentrations and of suicide, we undertook a detailed meta-analysis of completed suicides in relation to 5 major pollutants over 6 years in the 16 administrative regions of the Republic of Korea, while also controlling for other established influences on suicide rates. Of the 5 major pollutants examined, ozone concentrations had a powerful association with suicide rate, extending back to 4 weeks. Over the range of 2 standard deviations (SD) around the annual mean ozone concentration, the adjusted suicide rate increased by an estimated 7.8% of the annual mean rate. Particulate matter pollution also had a significant effect, strongest with a 4-week lag, equivalent to 3.6% of the annual mean rate over the same 2 SD range that approximated the half of annual observed range. These results strongly suggest deleterious effects of ozone and particulate matter pollution on the major public health problem of suicide.

## Introduction

Suicide is a leading cause of death worldwide and increasingly recognized as a serious public health problem [1–2]. The adverse effects of air pollution on general health are well known: air pollution contributes to excess mortality and increased hospital admissions for respiratory and cardiovascular events [3–4]. Several studies have reported an association between suicide and air pollution. Air pollution is associated with increased emergency room visits for suicide attempts [5], and increased airborne concentration of particulate matter is associated with increased risk of completed suicide [6]. Also, a recent study in Taiwan found an influence of sulfur dioxide and ozone on suicide risk [7]. However, such reports have been inconsistent [6–7], possibly because these studies were conducted in limited geographic areas and did not consider some important covariates (e.g., celebrity suicide or economic variables) [8]. Here we report on a nationwide study in the Republic of Korea (ROK, population 50 million), testing for associations between 5 major air pollutants and suicide. Recognizing the wide geographic variability of air pollution, we conducted a meta-analysis of regional data, while also controlling for other established factors associated with suicide.

## Methods

### Regional suicide data

We obtained the daily number of completed suicide events in each of the 16 administrative regions of ROK over 6 years from January 1 2006 to December 31 2011. The data were thoroughly examined and verified by the Korea National Statistical Office (http://kostat.go.kr/portal/english). Data for those years were considered because contemporaneous daily regional air pollution data were available. Suicide data were extracted from death records defined as suicides according to the International Classification of Diseases-10 (ICD-10) codes X60-X84, which include suicides from all causes, including intentional self-poisoning and self-harm [8]. Average national weekly suicide numbers from January 2001 through December 2005 also were computed so as to allow adjustment for seasonal variation [9]. We used population and housing census data to calculate the suicide rate per 10 million persons in each region.

### Celebrity suicides

In order to control for the influence of celebrity suicides, we noted the periods following those events. We defined celebrity suicide as a suicide exposed during more than two weeks in news programs of the three major Korean national television networks (KBS, MBC and SBS) [8]. Eight suicides met the definition of celebrity suicide during the 6 years of this study. In addition, we defined the affected period as a month (30 days) after the first report of the celebrity suicide [8,10]. Time points within or partly within this 30-day window were coded 1, while all others were coded 0 on the celebrity variable.

### Air pollution, economic and meteorological data

Daily regional air pollution data were obtained from the Korean Ministry of Environment (http://www.airkorea.or.kr/airkorea/eng/) during the study period. They provided comprehensive data from 251 sites in 79 cities or areas nationwide. These data were grouped according to the 16 administrative regions of ROK (a mean of 15.7 sites per administrative region). Averaged values for each region were used in the analyses. Five major air pollution variables were considered: ozone, PM-10 (Particulate Matter, particulates with size of 10 μm in diameter or smaller), nitrogen dioxide, carbon monoxide and sulfur dioxide. The daily meteorological data (sunlight hours and temperature) [11] were obtained from the Korea Meteorological Administration (KMA, http://web.kma.go.kr/eng). The economic data [12] including consumer price index, unemployment rate, and stock index valuations (Korea Composite Stock Price Index, KOSPI), were extracted from the Korea National Statistical Office. The end-of-week and holiday closing values of the KOSPI were carried forward to the next active trading day. The most recent monthly data for the consumer price index and the unemployment rate were used each day.

### Ethics statement

Our research analyzes existing data that are publicly available in a manner that does not allow individual subjects to be identified; therefore ethics approval was deemed unnecessary.

### Statistical analysis

For data reduction, we averaged all daily variables in discrete weekly epochs. There were 313 epochs during the study period. All computations were performed using these binned weekly numbers. This data reduction step controlled for the known day-of-week effect on suicide rate [13].

We employed linear regression modeling to evaluate the association between five air pollution variables and suicide number in each of the sixteen regions. The regional weekly suicide rate per 10 million persons was considered as the dependent variable. Celebrity suicides, economic and meteorological variables were entered as covariates in the linear regression model. We included the regional weekly suicide rate per 10 million in the preceding week as a covariate to control for short-term trending of suicide, which we previously identified as a significant predictor of weekly suicide rate [8]. The average national monthly suicide number for the past 5 years by month matching each weekly data set also was entered as a covariate in order to control for seasonality. We examined the air pollutant data lagged weekly by up to 6 weeks preceding the suicide events (time lag 0 through time lag 6). The presence of statistical heterogeneity was assessed with the I-square heterogeneity test and Cochran’s Q test [14]. Then, these regional results were meta-analyzed with the DerSimonian-Laird random effect model which accounts for heterogeneity among meta-analyzed studies [15] using Metasoft software [16]. We present the outputs of the meta-analysis as the regression coefficients (beta) and their standard deviations expressed as increased suicide numbers associated with unit increases of pollutant concentrations. Because multiple testing can raise type I error, *P* values of different time lags were controlled by Bonferroni’s correction. All statistical analyses were performed using the R 2.9.1 public statistics software (R package, http://www.r-project.org). Results were considered significant at a threshold of *P* < 0.05. The detailed definitions of the variables and formula of the linear regression model are given in S4 Table. All relevant data are within the Supporting Information file named S1 Data.

## Results

### Trend of suicide number and air pollution levels

Over the six years of the study (2006–2011), national suicide numbers trended upwards and showed seasonal variation with peaks in the spring months and troughs in the fall months (S1 Fig.). The average national weekly suicide rate per 10 million persons was 55.81 in the period, which corresponds to an average annual suicide rate of 29.1 per 100,000 persons per year. This rate for the ROK is one of the highest among OECD (Organization for Economic Co-operation and Development) nations [17]. The weekly suicide rate varied considerably by region (range 44.18 to 80.44—S1 Table). Trends of nationwide weekly average air pollutant levels are shown in S2 Fig. All five pollutants displayed marked variability from week to week and by region (S1 Table). For instance, the weekly average ozone concentration showed a 2-fold range across regions from 0.019 ppm (Seoul) to 0.037 ppm (Jeju). Temporal coefficients of variation of regional weekly ozone concentrations over the 6 years ranged from 0.28 to 0.45, with a national average of 0.33 (S1 Table). The annual range of concentrations of ozone was over 3-fold from trough to peak (S2A Fig.). However, these annual pollutant rhythms were not closely synchronized.

### The association between suicide rate and pollutant concentrations

**Fig 1:**
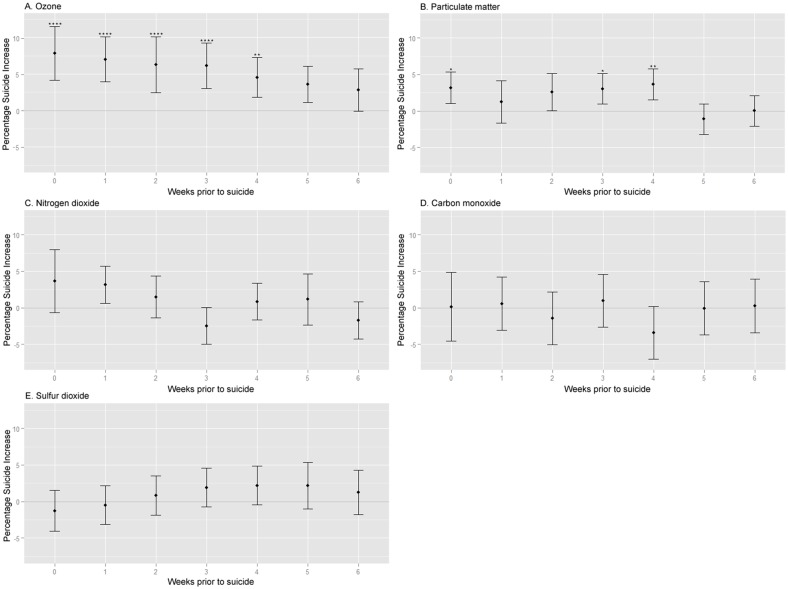
Suicide Increase Associated with Air Pollution Increase According To Weeks Prior to suicide. (*A*) Ozone; (*B*) Particulate matter; (*C*) Nitrogen dioxide; (*D*) Carbon monoxide; (*E*) Sulfur dioxide. *Corrected *P*< 0.05, **corrected *P*< 0.01, ***corrected *P*< 0.001, ****corrected *P*< 0.0001. Percentage suicide increase was calculated by multiplication of beta, range of pollutant concentration from-1SD to +1SD relative to the annual mean value (2 SD range) and inverse number of national weekly suicide rate per 10 million persons.

As the effect of the five pollutants was heterogeneous among different regions and different time points (I-square statistic >30 and Cochran’s Q test *P* < 0.05, see S2 Table for details), we performed random-effects meta-analysis (S2 Table). Fig. 1 shows the summary of meta-analyses of the effect of the five air pollutants with time lags from 0 to 6 weeks.

From time lag 0 to time lag 4, the level of ozone was significantly associated with suicide rate (Table 1). The greatest magnitude of effect was shown with time lag 0. The magnitude of this effect can be illustrated using the national average data: At time lag 0, for an increase in ozone concentration from-1SD to +1SD relative to the annual mean (an increase of 0.016 ppm) the weekly suicide rate increased by 4.37 per 10 million or 7.8% of the national weekly suicide rate of 55.81 per 10 million documented from January 2006 through December 2011 (suicide rate increase per 2 SD range ozone increase = 7.8%, 95% CI = 4.2%−11.5%, corrected *P* < 0.0001, meta-analysis of 16 linear regression analyses). This 2 SD range of ozone concentration corresponds to half of the observed annual range of ozone concentrations (S2A Fig.). As shown in Fig. 1A, the effect of ozone on suicide rate subsided gradually with increasing time lag.

**Table 1:** Associations of Pollutants with Averaged Weekly Suicide Rate per 10 million Persons from Lag 0 to Lag 4.

Pollutants	Weeks prior to suicide	Beta ^a^ ^.^	S.E.	% of Suicide Increase Relative to Annual Mean Rate for an Increase in Pollutant Concentration from-1SD to +1SD ^b^ ^.^	95% CI, Lower	95% CI, Upper	Corrected *P* ^c^ ^.^	I-square heterogeneity(%) ^d^ ^.^	Cochran’s Q’s *P* ^d^ ^.^
Ozone	0	274.01	65.61	7.8	4.2	11.5	<0.0001	42.77	0.04
Ozone	1	245.12	55.00	7.0	3.9	10.1	<0.0001	29.68	0.13
Ozone	2	220.28	68.38	6.3	2.5	10.1	<0.0001	54.99	0.004
Ozone	3	215.08	55.69	6.1	3.0	9.3	<0.0001	33.89	0.09
Ozone	4	158.15	48.46	4.5	1.8	7.2	<0.01	14.50	0.29
PM-10	0	0.047	0.016	3.2	1.0	5.3	0.03	0	0.89
PM-10	1	0.018	0.022	1.2	-1.7	4.1	1	37.04	0.07
PM-10	2	0.039	0.019	2.6	0.1	5.2	0.31	22.18	0.20
PM-10	3	0.045	0.016	3.0	0.9	5.1	0.03	0	0.91
PM-10	4	0.054	0.016	3.6	1.5	5.7	<0.01	0	0.90

Table 1. are results of meta-analyses of regional data in South Korea from 2006 through 2011.

Abbreviations: S.E., Standard Error; SD, Standard Deviation; CI, Confidence Interval; PM-10, Particulate Matter (particulates with size of 10 μm in diameter or smaller).

^a.^ Increased weekly suicides per 10 million persons when the level of air pollution increases by 1 unit.

^b.^ Calculated by multiplication of beta, 2 SD range of national level of air pollution and inverse number of national weekly suicide rate per 10 million persons.

^c.^ Corrected by Bonferroni’s method for the tests of the number of time lags.

^d.^ I-square heterogeneity test and Cochran’s Q test were employed for testing the presence of statistical heterogeneity in meta-analyses.

The PM-10 level also had significant associations with the weekly suicide rates in time lags 0, 3 and 4 (corrected *P* = 0.03 for time lag 0, *P* = 0.03 for time lag 3, and *P* < 0.01 for time lag 4, Table 1). As the PM-10 increased by 1 unit (1 μg/m^3^), weekly suicides per 10 million persons increased by 0.047 in time lag 0. Using the national average data, for an increase in PM-10 concentration from-1SD to +1SD relative to the annual mean (an increase of 37.82 μg/m^3^) the weekly suicide rate increased by 1.76 per 10 million or 3.2% of the national weekly suicide rate of 55.81 per 10 million at time lag 0. This 2 SD range of PM-10 concentration (32.72–70.54μg/m^3^) corresponds approximately to half of the observed annual range of PM-10 concentrations (S2B Fig.). This association disappeared at time lags 1 and 2. The most prominent effect of PM-10 occurred with a four week interval, when the effect corresponded to a weekly suicide rate increase of 2.03 per 10 million or 3.6% of the national weekly suicide rate of 55.81 per 10 million (suicide rate increase per 2 SD range PM-10 increase = 3.6%, 95% CI = 1.5%−5.7%, corrected *P* < 0.01, meta-analysis of 16 linear regression analyses).

Regional plots (S3 Fig.) and forest plots (S4 Fig.) for ozone and PM-10 in their most prominent associated time lag (lag 0 for ozone, lag 4 for PM-10) are presented. We found regional differences in the association between air pollutants and suicide rates (S3 Fig.). A notable finding is that the three providences with high suicide rate (Chungnam, Gangwon and Chungbuk) showed the largest increases of suicide associated with a 2 SD increase of ozone concentration (S4A Fig.).

We found no significant associations for nitrogen dioxide, carbon monoxide and sulfur dioxide in meta-analyses of all time lags. All results of linear regression modeling in 16 regions with 7 different time lags and their meta-analyses are provided in S2 and S3 Tables.

## Discussion

In this nationwide study, we found that increasing concentrations of ozone and atmospheric particulate matter were related to suicide rate. Of all 5 pollutants we examined, ozone had the strongest associations with suicide rate, extending back to 4 weeks before the suicide events. Increase of ozone concentration over a 2 SD range (0.016 ppm) that approximated half of the observed annual range was associated with a 7.8% increase of weekly suicide rate relative to the annual mean weekly suicide rate. Increase of PM-10 concentration over a 2 SD range (37.82μg/m^3^) that also approximated half of the observed annual range was associated with a 3.2% to 3.6% increase of weekly suicide rate at time lags 0 and 4, respectively.

There have been previous studies that suggest multiple mechanisms for the effect of air pollution on the central nervous system (CNS). First, air pollution can affect the immune system and thereby induce behavioral changes through effects on neurotransmitter systems [18–19]. Ozone can induce inflammation in the lungs of exposed subjects [20], and this peripheral inflammation could impact on the CNS through circulating cytokines [21]. In addition, exposure to particulate matter for several weeks increases proinflammatory cytokines in mouse brain [22]. Furthermore, exposure to particulate matter has been associated with hypomethylation of the gene for inducible nitric oxide synthase (*iNOS*), which regulates a key step during inflammatory reactions [23]. Considering that the immune system could affect the development of depression [24], the reported effects of ozone and particulate matter on cytokines may be relevant to our observation on suicide rates. Second, stress hormones could be linked to air pollution and suicide. According to Errol et al., brief exposure of rats to ozone and particulate matter results in activation of the hypothalamo-pituitary-adrenal (HPA) axis [25]. Continuous exposure to air pollutants could cause HPA axis dysregulation, which is associated with the pathobiology of suicide in mood disorder [26]. Third, ozone or its reaction products could influence the metabolism of serotonin [27], one of the neurotransmitters associated with aggressive behavior and suicide [28–30]. These previous findings are relevant to our observed strong association of ozone concentration with suicide rate. Meanwhile, air pollution can influence suicide indirectly. For example, exposure to air pollutants aggravates respiratory disease [3] and increases the risk of depressive episodes among individuals with pre-existing cardiovascular disease or diabetes mellitus [31]. In addition, exposure to air pollutants is associated with a high incidence of spontaneous abortion which is a risk factor for depression [32]. These physical burdens could increase the risk of suicide in the population [33–34]. In our results, the association between PM-10 and suicide rate was observed at time lag 0, however, it was absent at time lags 1 and 2. We speculated that the effects of disease aggravations, bereavement or increase of physical burdens on suicide rate require intervals to link with consequent suicide events. Further studies with individual risks of suicide such as psychiatric or medical disease could be helpful to clarify this hypothesis.

In prior research, Yang et al, found that air pollutants such as sulfur dioxide and ozone influence the risk of suicide over longer time scales [7]. Also, Kim et al, have reported that transient increases of particulate matter concentration are related to suicide risk [6]. In addition, a German study conducted by Biermann et al, reported an association between ozone level and completed suicide.[35] However, those studies had some limitations. First, their results were limited to the data from metropolitan areas, and data from rural areas were excluded. Since the suicide rates vary by region [36], region-specific studies risk confounding by Type I error (false positive). Our analyses also suggest that the level of association between air pollution and suicide varies among regions (S1 Table, S3 and S4 Figs.), supporting the possibility of non-generalizable findings in prior studies. In addition, the earlier studies did not consider multiple other variables that affect suicide risk. For instance, celebrity suicides have a marked effect on the national suicide rate [8,10,37]. Economic factors, including consumer price index, unemployment rate, and stock index valuations, also are well known for their association with suicide rates [12,38]. In addition, meteorological factors, like sunlight hours and temperature, have established associations with suicide rate [39].

To complement these prior studies, we performed a nationwide regional meta-analysis while controlling for important covariates like celebrity suicide, economic factors, and meteorological factors, as well as seasonality of suicide rates. We also controlled for short term trending as revealed by our previous report [8]. For this purpose we included the previous week’s suicide rate as a covariate. In addition, we examined multiple time periods from 0 to 6 weeks prior to the day of suicide, to test for immediate and delayed effects of pollutants. Thus, this study is the first nationwide analysis assessing the proximate and near-term delayed effects of air pollution on suicide rate.

As a result of pollution control policy in all countries of the world, sulfur dioxide concentrations, which result from fossil fuel combustion, have been significantly reduced [3]. That may be a factor in our finding of no significant association between ambient sulfur dioxide concentration and suicide. Consequently, other air pollutants like ozone and particulate matter which are less related to fossil fuels, have received increasing attention recently [3]. Guidelines issued by the World Health Organization (WHO) aim at reduction of ozone and particulate matter because of their association with overall mortality [40]. Our results showing strong associations of ozone and PM-10 concentrations with suicide add to the public health urgency for reduction of these pollutants.

A limitation of our study is retrospective design based on national databases, so that we could not test the effect of other recognized air pollutants such as PM-2.5 (Particulate Matter, particulates with size of 2.5 μm in diameter or smaller) [5], lead, ammonia, radioactive pollutants or volatile organic compounds. There is also a possibility that ozone, a highly reactive molecule, could exert its effects through secondary reaction products. Further studies with expanded data will be required.

As previously mentioned, the strength of associations between suicide rates and pollutant concentrations varied among regions. Factors influencing these differences could be analyzed in further studies. Nevertheless, the overall meta-analysis revealed a highly significant association of suicide rate with ozone concentration, as well as a strong association with particulate matter pollution. In conclusion, our analysis expands the evidence for a link between suicide and air pollutants through this regional meta-analysis. Our data direct attention especially to ozone and particulate matter as the significant drivers of this association.

## Supporting Information

S1 Data Data File. (XLSX)

S1 Fig Trend of weekly national suicide number per 10 million persons in Korea. (TIF)

S2 Fig Trend of weekly average air pollution levels in Korea. (TIF)

S3 Fig Regional plots for ozone and PM-10 in their most prominent associated time lag (lag 0 for ozone; lag 4 for PM-10). (TIF)

S4 Fig Forest plots for ozone and PM-10 in their most prominent associated time lag (lag 0 for ozone; lag 4 for PM-10). (TIF)

S1 Table Weekly suicide rate and weekly averages of air pollution levels in 16 regions of South Korea 2006 through 2011. (DOCX)

S2 Table Meta-analyses of linear regression modeling in 16 regions with 7 different time lags. (XLSX)

S3 Table Linear regression modeling in 16 regions with 7 different time lags. (XLSX)

S4 Table Variables included in the analysis and their detailed descriptions. (DOCX)
