# Supplementary figures and images for: Association between Air Pollution and Suicide in South Korea: A Nationwide Study
Source: PLoS One. 2015 Feb 18;10(2):e0117929. doi: 10.1371/journal.pone.0117929 (PMC4333123; doi:10.1371/journal.pone.0117929)

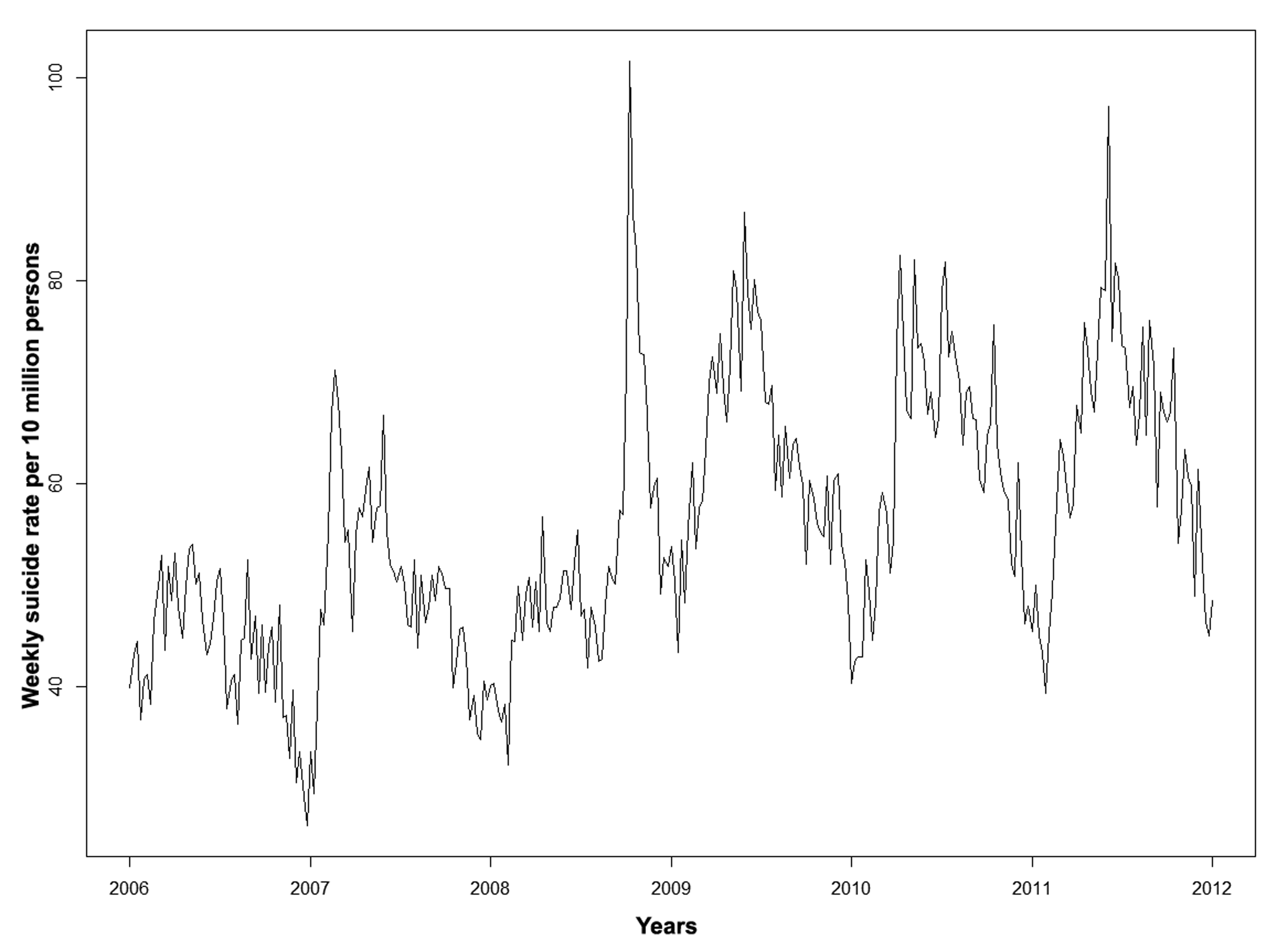

Supplement: S1 Fig — (TIF) [file pone.0117929.s002.tif]

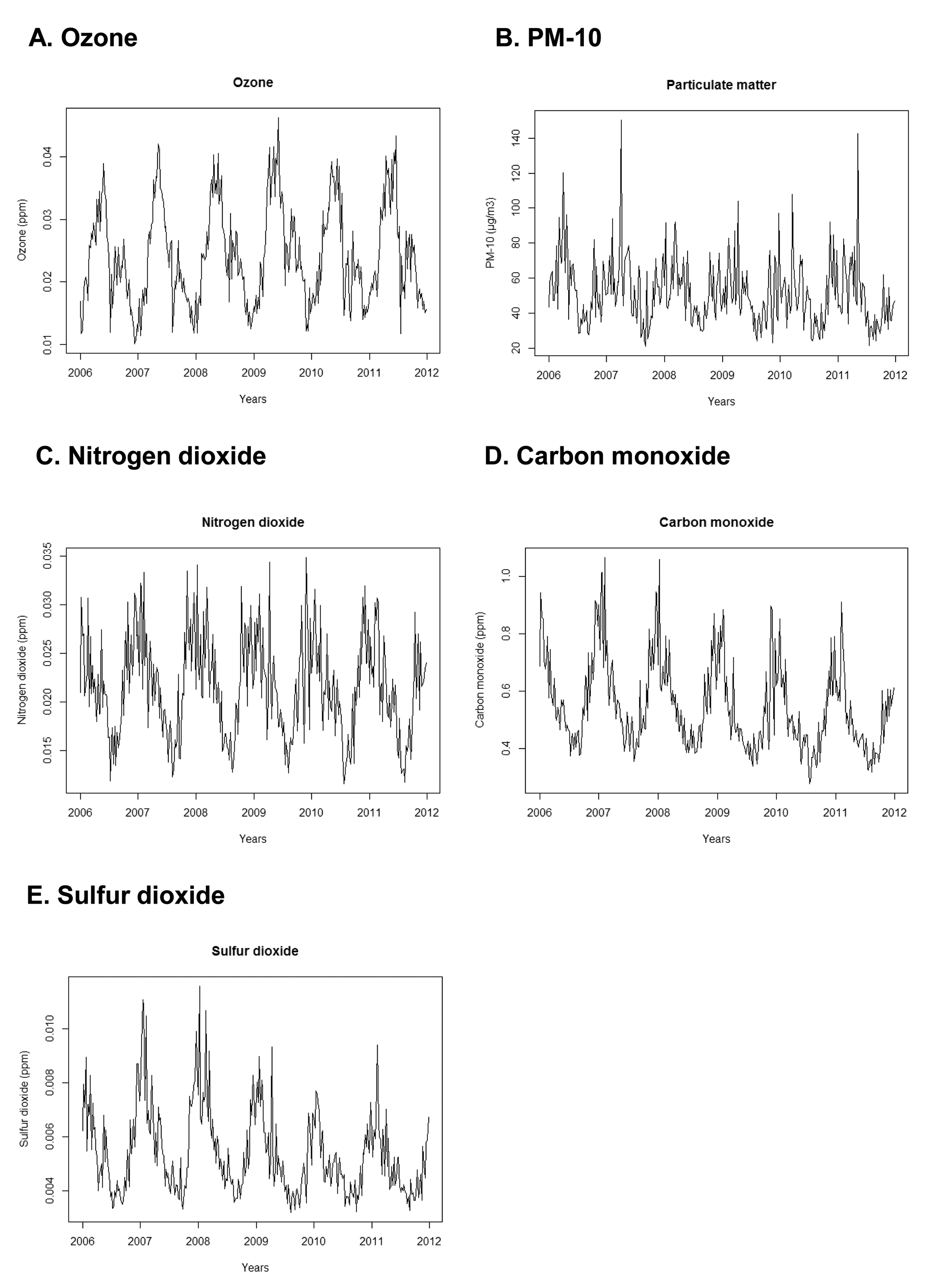

Supplement: S2 Fig — (TIF) [file pone.0117929.s003.tif]

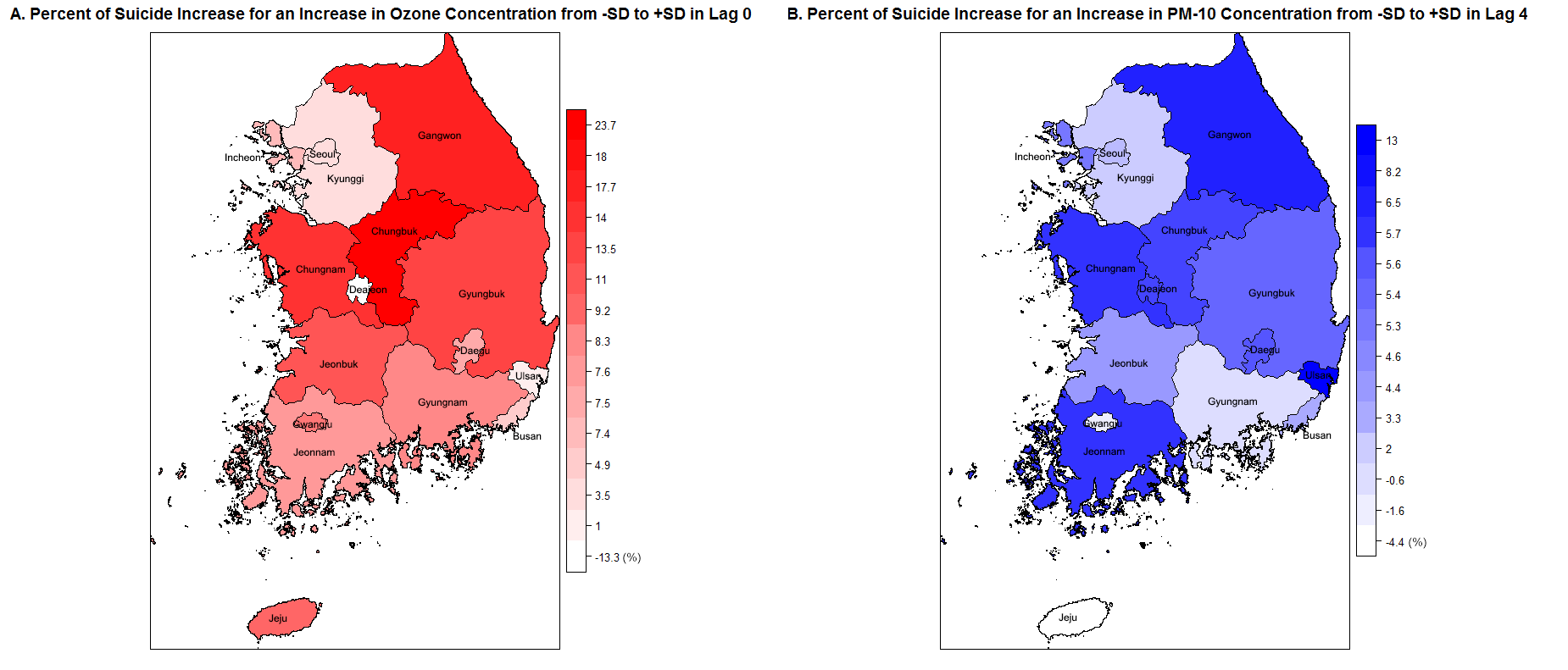

Supplement: S3 Fig — (TIF) [file pone.0117929.s004.tif]

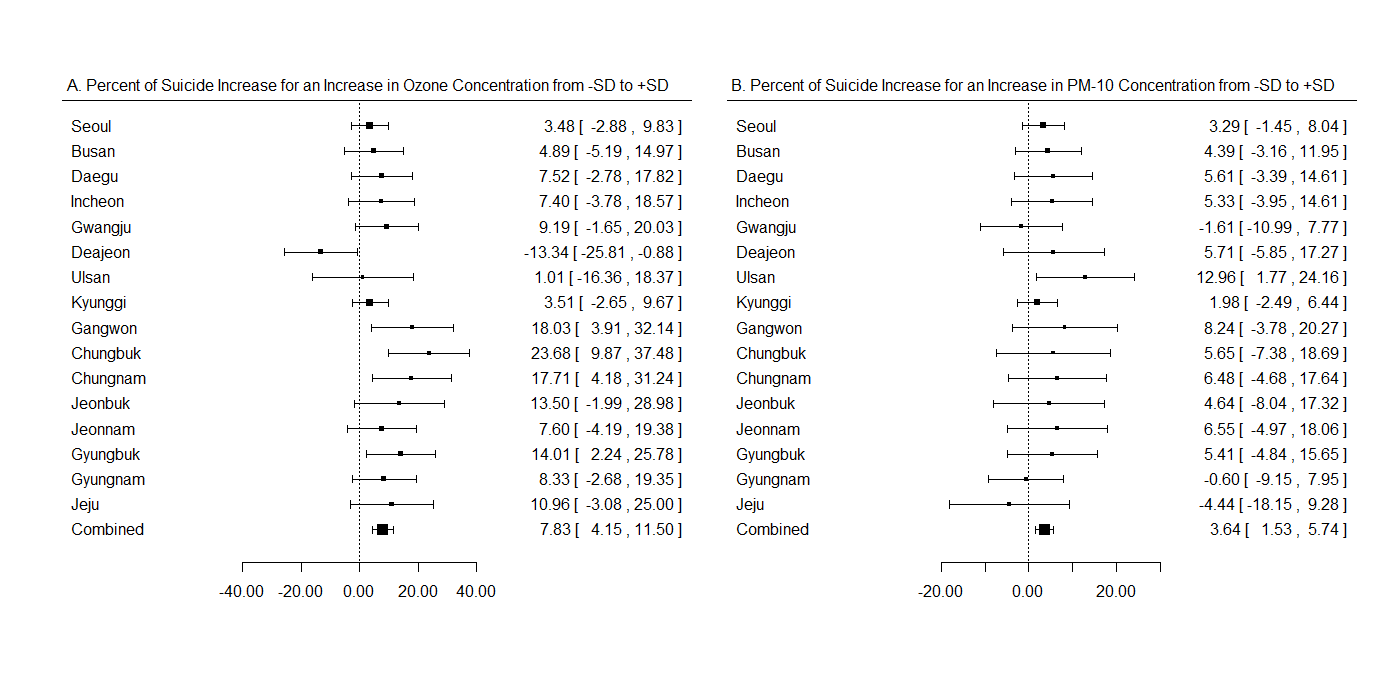

Supplement: S4 Fig — (TIF) [file pone.0117929.s005.tif]
