# Supplementary material for: Association between Air Pollution and Suicide in South Korea: A Nationwide Study
Source: PLoS One. 2015 Feb 18;10(2):e0117929. doi: 10.1371/journal.pone.0117929 (PMC4333123; doi:10.1371/journal.pone.0117929)
Supplement: S1 Table — (DOCX) [file pone.0117929.s006.docx]

**Table S1. Weekly Suicide Rate and Weekly Averages of Air pollution Levels in 16 Regions of South Korea 2006 through 2011.**

| Region | Weekly  Suicide rate  per 10 million | Ozone (O_3_, ppm) | | PM-10 (μg/m^3^) | | Nitrogen dioxide (NO_2_, ppm) | | Carbon monoxide (CO, ppm) | | Sulfur dioxide (SO_2_, ppm) | |
| --- | --- | --- | --- | --- | --- | --- | --- | --- | --- | --- | --- |
|  |  | Mean (±SD) | CV | Mean (±SD) | CV | Mean (±SD) | CV | Mean (±SD) | CV | Mean (±SD) | CV |
| Seoul | 46.18 | 0.019 (±0.008) | 0.43 | 54.36 (±22.78) | 0.42 | 0.036 (±0.009) | 0.24 | 0.59 (±0.19) | 0.32 | 0.006 (±0.002) | 0.34 |
| Busan | 56.52 | 0.026 (±0.007) | 0.28 | 52.03 (±19.23) | 0.37 | 0.021 (±0.005) | 0.24 | 0.41 (±0.09) | 0.22 | 0.006 (±0.001) | 0.25 |
| Daegu | 51.11 | 0.022 (±0.010) | 0.42 | 51.18 (±19.87) | 0.39 | 0.024 (±0.007) | 0.30 | 0.57 (±0.16) | 0.28 | 0.006 (±0.002) | 0.40 |
| Incheon | 56.75 | 0.022 (±0.008) | 0.35 | 59.86 (±21.87) | 0.37 | 0.030 (±0.007) | 0.25 | 0.60 (±0.18) | 0.29 | 0.007 (±0.002) | 0.27 |
| Gwangju | 46.52 | 0.024 (±0.009) | 0.39 | 48.56 (±21.63) | 0.45 | 0.021 (±0.007) | 0.32 | 0.56 (±0.18) | 0.32 | 0.004 (±0.001) | 0.38 |
| Deajeon | 51.58 | 0.021 (±0.009) | 0.44 | 45.82 (±19.15) | 0.42 | 0.021 (±0.006) | 0.31 | 0.59 (±0.22) | 0.38 | 0.004 (±0.002) | 0.42 |
| Ulsan | 44.18 | 0.023 (±0.007) | 0.29 | 50.76 (±20.22) | 0.40 | 0.023 (±0.005) | 0.22 | 0.46 (±0.12) | 0.25 | 0.008 (±0.002) | 0.30 |
| Kyunggi | 52.78 | 0.020 (±0.008) | 0.41 | 61.34 (±22.73) | 0.37 | 0.030 (±0.007) | 0.25 | 0.62 (±0.20) | 0.32 | 0.006 (±0.002) | 0.34 |
| Gangwon | 79.47 | 0.025 (±0.009) | 0.37 | 53.77 (±23.13) | 0.43 | 0.018 (±0.005) | 0.28 | 0.66 (±0.25) | 0.38 | 0.005 (±0.003) | 0.58 |
| Chungbuk | 69.35 | 0.023 (±0.010) | 0.45 | 59.62 (±22.32) | 0.37 | 0.021 (±0.006) | 0.30 | 0.69 (±0.33) | 0.48 | 0.006 (±0.004) | 0.60 |
| Chungnam | 80.44 | 0.024 (±0.008) | 0.36 | 48.49 (±19.43) | 0.40 | 0.017 (±0.005) | 0.28 | 0.58 (±0.16) | 0.27 | 0.004 (±0.001) | 0.34 |
| Jeonbuk | 64.95 | 0.023 (±0.008) | 0.35 | 53.39 (±18.88) | 0.35 | 0.015 (±0.004) | 0.28 | 0.53 (±0.15) | 0.28 | 0.005 (±0.001) | 0.26 |
| Jeonnam | 59.76 | 0.028 (±0.009) | 0.30 | 43.62 (±16.73) | 0.38 | 0.017 (±0.004) | 0.24 | 0.49 (±0.12) | 0.25 | 0.007 (±0.002) | 0.22 |
| Gyungbuk | 62.70 | 0.025 (±0.008) | 0.34 | 51.35 (±17.98) | 0.35 | 0.019 (±0.006) | 0.29 | 0.63 (±0.23) | 0.37 | 0.006 (±0.002) | 0.40 |
| Gyungnam | 60.20 | 0.026 (±0.008) | 0.31 | 47.46 (±18.16) | 0.38 | 0.020 (±0.005) | 0.27 | 0.43 (±0.12) | 0.27 | 0.005 (±0.001) | 0.30 |
| Jeju | 57.34 | 0.037 (±0.010) | 0.28 | 44.47 (±21.93) | 0.49 | 0.011 (±0.003) | 0.32 | 0.33 (±0.09) | 0.27 | 0.003 (±0.001) | 0.44 |
| Total | 55.81 | 0.024 (±0.008) | 0.33 | 51.63 (±18.91) | 0.37 | 0.022 (±0.005) | 0.24 | 0.55 (±0.15) | 0.28 | 0.005 (±0.002) | 0.29 |

Abbreviations: PM-10, Particulate Matter (particulates with size of 10 µm in diameter or smaller); SD, Standard Deviation; CV, Coefficient of Variation (=SD/mean).
