# Supplementary material for: Association between Air Pollution and Suicide in South Korea: A Nationwide Study
Source: PLoS One. 2015 Feb 18;10(2):e0117929. doi: 10.1371/journal.pone.0117929 (PMC4333123; doi:10.1371/journal.pone.0117929)
Supplement: S4 Table — (DOCX) [file pone.0117929.s009.docx]

**Table S4. Variables included in the analysis^1^ and their detailed descriptions**

| **Variable** | **Description** |
| --- | --- |
| **Dependent variable** |  |
| Suicide *_w,r_* | Weekly suicide rate per 10 million persons in week (*w*) by region (*r*) |
| **Independent variables** |  |
| Ozone *_w,r_* | Weekly average of daily ozone concentration in week (*w*) by region (*r*). |
| PM-10 *_w,r_* | Weekly average of daily concentration of particulates with size of 10 µm in diameter or smaller in week (*w*) by region (*r*). |
| NO2 *_w,r_* | Weekly average of daily nitrogen dioxide concentration in week (*w*) by region (*r*). |
| CO *_w,r_* | Weekly average of daily carbon monoxide concentration in week (*w*) by region (*r*). |
| SO2 *_w,r_* | Weekly average of daily sulfur dioxide concentration in week (*w*) by region (*r*). |
| Temp *_w,r_* | Weekly average of average daily temperature in week (*w*) by region (*r*). |
| Sunlight *_w,r_* | Weekly average daily sunlight duration in week (*w*) by region (*r*). |
| KOSPI *_w_* | Weekly average of daily Korea Composite Stock Price Index (KOSPI) by week (*w*). The end-of-week and holiday closing values of the KOSPI were carried forward to the next active trading day. |
| Unemployment *_w_* | Most recent monthly unemployment rate preceding each week (*w*). |
| Price *_w_* | Most recent monthly consumer price index preceding each week (*w*). |
| Average *_w_* | Average national monthly suicide number for past 5 years by month matching each weekly data set (*w*). ^2^ |
| Celebrity *_w_* | Week (*w*) within or partly within 30-days after the first report of the celebrity suicide were coded 1, while all others were coded 0 on the celebrity variable. |
| Suicide *_w-t-1,r_* | Weekly suicide rate per 10 million persons in preceding week (*w-t-1*) by region (*r*) |

^1^ Formula of linear regression model of time lag *t*

$$\check{\mathrm{Suicide}_{w,r}}=\check{\beta}_{0, r,t}+\check{\beta}_{1, r,t}\mathrm{Ozone}_{w-t,r}+\check{\beta}_{2, r,t}{\mathrm{PM}­10}_{w-t,r}+\check{\beta}_{3, r,t}{NO2}_{w-t,r}+\check{\beta}_{4, r,t}\mathrm{CO}_{w-t,r}+\check{\beta}_{5, r,t}{SO2}_{w-t,r}+ \check{\beta}_{6, r,t}\mathrm{Temp}_{w,r}+\check{\beta}_{7, r,t}\mathrm{Sunlight}_{w,r}+ \check{\beta}_{8, r,t}\mathrm{KOSPI}_{w}+ \check{\beta}_{9, r,t}\mathrm{Unemployment}_{w}+ \check{\beta}_{10, r,t}\mathrm{Price}_{w}+ \check{\beta}_{11, r,t}\mathrm{Average}_{w}+ \check{\beta}_{12, r,t}\mathrm{Celebrity}_{w}+\check{\beta}_{13, r,t}\mathrm{Suicide}_{w-t-1,r}$$

*r* = 1 (Seoul), 2 (Busan), 3 (Daegu), 4 (Incheon), 5 (Gwangju), 6 (Deajeon), 7 (Ulsan), 8 (Kyunggi), 9 (Gangwon), 10 (Chungbuk), 11 (Chungnam), 12 (Jeonbuk), 13 (Jeonnam), 14 (Gyungbuk), 15 (Gyungnam), 16 (Jeju)

*w* = 1 (2006-01-01 ~ 2006-01-07), 2 (2006-01-08 ~ 2006-01-14) … 313 (2011-12-25 ~ 2011-12-31)

*t* = 0, 1, 2 … 5, 6

Then, the regression coefficients ($\check{\beta}_{1, r,t}$,$\check{\beta}_{2, r,t}$, $\check{\beta}_{3, r,t}$, $\check{\beta}_{4, r,t}$, $\check{\beta}_{5, r,t}$) and their standard errors were used for meta-analysis of each time lag.

^2^ For example, in 2010, the average national monthly suicide number for 2005-2009 were used for seasonality adjustment.
